# A systematic review and narrative synthesis of data-driven studies in schizophrenia symptoms and cognitive deficits

**DOI:** 10.1038/s41398-020-00919-x

**Published:** 2020-07-21

**Authors:** Tesfa Dejenie Habtewold, Lyan H. Rodijk, Edith J. Liemburg, Grigory Sidorenkov, H. Marike Boezen, Richard Bruggeman, Behrooz Z. Alizadeh

**Affiliations:** 1 Department of Epidemiology, University Medical Center Groningen, University of Groningen, Groningen, The Netherlands; 2 Department of Psychiatry, Rob Giel Research Center, University Medical Center Groningen, University Center for Psychiatry, University of Groningen, Groningen, The Netherlands; 3 Department of Pediatric Surgery, University Medical Center Groningen, University of Groningen, Groningen, The Netherlands; 4 Department of Neuroscience, University Medical Center Groningen, University of Groningen, Groningen, The Netherlands

**Keywords:** Long-term memory, Scientific community, Schizophrenia

## Abstract

To tackle the phenotypic heterogeneity of schizophrenia, data-driven methods are often applied to identify subtypes of its symptoms and cognitive deficits. However, a systematic review on this topic is lacking. The objective of this review was to summarize the evidence obtained from longitudinal and cross-sectional data-driven studies in positive and negative symptoms and cognitive deficits in patients with schizophrenia spectrum disorders, their unaffected siblings and healthy controls or individuals from general population. Additionally, we aimed to highlight methodological gaps across studies and point out future directions to optimize the translatability of evidence from data-driven studies. A systematic review was performed through searching PsycINFO, PubMed, PsycTESTS, PsycARTICLES, SCOPUS, EMBASE and Web of Science electronic databases. Both longitudinal and cross-sectional studies published from 2008 to 2019, which reported at least two statistically derived clusters or trajectories were included. Two reviewers independently screened and extracted the data. In this review, 53 studies (19 longitudinal and 34 cross-sectional) that conducted among 17,822 patients, 8729 unaffected siblings and 5520 controls or general population were included. Most longitudinal studies found four trajectories that characterized by stability, progressive deterioration, relapsing and progressive amelioration of symptoms and cognitive function. Cross-sectional studies commonly identified three clusters with low, intermediate (mixed) and high psychotic symptoms and cognitive profiles. Moreover, identified subgroups were predicted by numerous genetic, sociodemographic and clinical factors. Our findings indicate that schizophrenia symptoms and cognitive deficits are heterogeneous, although methodological limitations across studies are observed. Identified clusters and trajectories along with their predictors may be used to base the implementation of personalized treatment and develop a risk prediction model for high-risk individuals with prodromal symptoms.

## Introduction

In psychiatry, phenotypic heterogeneity of disorders and their overlapping symptoms that may presumably share some fundamental biologic underpinnings is a major challenge for tailoring individualized therapies^1^. Similarly, the course and phenotypic expression of schizophrenia are variable^2^. Schizophrenia is a complex polygenic psychotic disorder with a lifetime morbidity risk of 0.7%^3^. The twin- and SNP-based heritability estimate of schizophrenia was 80%^4^ and 30%^5^, respectively.

According to the diagnostic and statistical manual of mental disorders (DSM) criteria, the clinical manifestations of schizophrenia are positive (e.g., hallucinations, delusions and disorganized behaviour) and negative (e.g., emotional expressive deficit, social amotivation, social withdrawal and difficulty in experiencing pleasure) symptoms^6^. Cognitive deficit is also one of the hallmark manisfestations of schizophrenia that occur in 75–80% of patients and often associated with poor daily functioning and quality of life^7^. Cognitive impairment in schizophrenia can be selective or general though the most common deficits occur in executive function, processing speed, memory (e.g. episodic, verbal and working), attention, verbal fluency, problem-solving and social cognition^8–11^. Patients harbor a wide range of subjectively defined symptoms, which together yields instinctively heterogeneous groups of people who are collectively diagnosed with schizophrenia. Subclinical or prodromal symptoms are also evident in relatives of patients with schizophrenia and healthy general population^12–14^.

Despite a century of efforts, understanding the heterogeneity in the clinical presentation and course of schizophrenia has been unsuccessful. This can be due to the subjective measurement of its clinical symptoms, variation in response to treatment, lack of valid, stable, and meaningful sub-phenotyping methods, and molecular complexity with limited understanding of the pathophysiology^15–17^. Phenotypic heterogeneity can be related to several intrinsic and extrinsic factors and expressed in patients, time, and disease sub-phenotypes^16,18^. Identification of meaningful homogeneous subgroups of people based on their symptoms or endophenotypes (e.g. neuropsychological markers, neural substrates, and neurological soft signs) requires the use of both supervised and unsupervised analyses. Distinguishing heterogeneous patients to more behaviorally and biologically similar subgroups is expedient not only to unveil common etiologies but also to examine the patterns of clinical symptoms, understand the biology of disease, predict treatment response and develop a new targeted treatment that improves recovery and functional outcomes^15,16,19,20^.

For tackling heterogeneity, in the past decade, numerous efforts have been undertaken by carefully designing studies and developing statistical models implemented in various programming languages and software^16^. In 2013, the American Psychiatric Association also endorsed a dimensional approach to identify intermediate categories based on the subjective report of severity of symptoms^6^. As a result, researchers have been using latent class cluster analyses and growth mixture models to explore clusters of individuals and trajectories of clinical symptoms in various settings^15,21,22^. Statistical methods can be used to identify subgroups and describe within and between individual variations to guide clinicians and statisticians to explore the relationship of diseases with various clinical and functional outcomes, treatment response, and neuropathological change. Moreover, subtyping using imaging, biological and symptom data is a recognizable method and widely used in psychiatric research^21^.

Several reviews have been conducted on positive symptoms^23^, negative symptoms^24–26^ and cognitive dysfunction^7,9,27–35^. However, these reviews have largely focused on the conventional approach for determining an average change in the course of symptoms over time and the difference between subjects (e.g., patient vs sibling, sibling vs control, or patient vs control) and diagnosis. Reviewed studies are also based on correlation analysis, which is believed not to be a strong measure of association between predictors and outcomes^36^. Besides, these primary studies vary in terms of study population and use of assessment tools, scoring and standardization techniques, and have several limitations, such as small sample size, short duration of follow-up and limited use of data from healthy siblings and/or controls^9,37,38^. Of interest, none of these reviews fully addressed evidence from both longitudinal and cross-sectional data-driven studies on schizophrenia symptoms and cognitive deficits among patients with schizophrenia spectrum disorders, relatives and healthy controls. Taken together, thus far, our understanding of the heterogeneity of the course of schizophrenia symptoms and cognitive deficits is still limited. In the present systematic review, we summarized the contemporary evidence from cross-sectional and longitudinal studies on positive and negative symptoms and cognitive deficits among patients with schizophrenia spectrum disorders, their unaffected siblings and healthy people. Additionally, we explored the extent and origin of heterogeneity across studies. We further highlighted common methodological gaps and point out future directions to optimize the translatability of evidence from data-driven studies within the outlook of a personalized approach.

## Methods

### Registration and reporting

This systematic review was conducted and reported based on a registered protocol^39^ and the Preferred Reporting Items for Systematic Review and Meta-Analysis (PRISMA) statement (Supplementary File 1), respectively^40,41^. The screening and selection process of the reviewed articles are further illustrated using a PRISMA flow diagram.

### Databases and search terms

A systematic search of PubMed, PsycINFO, PsycTESTS, PsycARTICLES, SCOPUS, EMBASE and Web of Science electronic databases was performed. A comprehensive search strategy was developed for PubMed and adapted for each database in consultation with a medical information specialist (Supplementary File 2). The following search terms were used in their singular or plural form in the title, abstract, keywords and text fields of the articles: “schizophrenia”, “psychosis”, “non-affective psychosis”, “cognitive deficit”, “cognitive dysfunction”, “cognitive alteration”, “negative symptoms”, “deficit syndrome”, “positive symptoms”, “psychopathology”, “cognit*”, “neuropsycholog*”, “neurocognition”, “longitudinal”, “follow-up”, “course”, “heterogeneity”, “endophenotype”, “profile”, “cluster analysis”, “siblings”, “healthy controls”, “latent class analyses”, “Symptom trajectories”, “traject*”, “group modelling” and “trajectory”. Cross-references of included articles and grey literature were also hand-searched. Furthermore, we searched the table of contents of the journals of Schizophrenia Research, Schizophrenia Bulletin, Acta Psychiatrica Scandinavica, BMC Psychiatry, American Journal of Psychiatry and British Journal of Psychiatry to explore relevant studies. The freezing date for the final search was August 2019. In this review, we use ‘trajectory’ for groups identified in longitudinal studies and “cluster” for groups identified in cross-sectional studies.

### Inclusion and exclusion criteria

Studies which met the following criteria were included: (1) longitudinal and cross-sectional studies; (2) studies that reported at least two clusters or trajectory groups of individuals using a statistical method based on a distinct positive symptom, negative symptom, and cognitive deficit or a combination of these symptoms; (3) studies conducted in patients with schizophrenia spectrum disorders, unaffected relatives, or healthy individuals irrespective of their clinical (e.g. medication status, severity of illness) and sociodemographic characteristics; and (4) studies published in English from 2008 to 2020. The publication year was limited to the last decade to capture the latest available evidence, which is likely to provide statistically powerful estimates and successfully subtyping schizophrenia symptoms given the increased number of large cohorts. To maximize the number of searched articles, the follow-up period in longitudinal studies was not restricted. Longitudinal studies based on the analyses of the mean levels of change of symptom scores were excluded because they did not capture individuals’ patterns of change over time by treating between-subject variation as an error, so that the actual heterogeneity of groups cannot be revealed^42^. Also, studies based on the non-statistical methods of clustering (e.g. family-based clustering) were excluded. Review papers, commentaries, conference abstracts, duplicate studies, editorials, and qualitative studies were excluded as well. Furthermore, we excluded studies in which the trajectory groups or clusters were generated based on scores constructed using a combination of schizophrenia symptoms and other unspecified psychotic symptoms.

### Data retrieval and synthesis

Studies retrieved from all databases were exported to RefWorks version 2.0 for Windows web-based citation manager, which followed by the removal of close and exact duplicates. All independent studies were exported to a Microsoft Excel spreadsheet to screen for further inclusion criteria. Authors T.D.H. and L.H.R. independently screened the titles and abstracts. The two reviewers had a substantial agreement (Kappa statistic (*κ*) = 0.62). Inconsistent decisions were discussed and solved with consensus. Finally, full-text was reviewed, and the following data were independently extracted by T.D.H. and L.H.R.: first author name, publication year, country, cohort/research center, study population, sample size, symptom dimension(s), assessment tool, study design, duration of follow-up for longitudinal studies, frequency of assessment, method of calculating composite score, method of clustering/trajectory analysis, number of identified clusters or trajectory groups and significant correlates of clusters and predictors of trajectories^43^. The corresponding author was contacted by email if the full-text of included article was not accessible. When studies did not report the cohort or research center, we extracted the institutional affiliation of the first or corresponding author.

## Results

### Search results

**Fig. 1:**
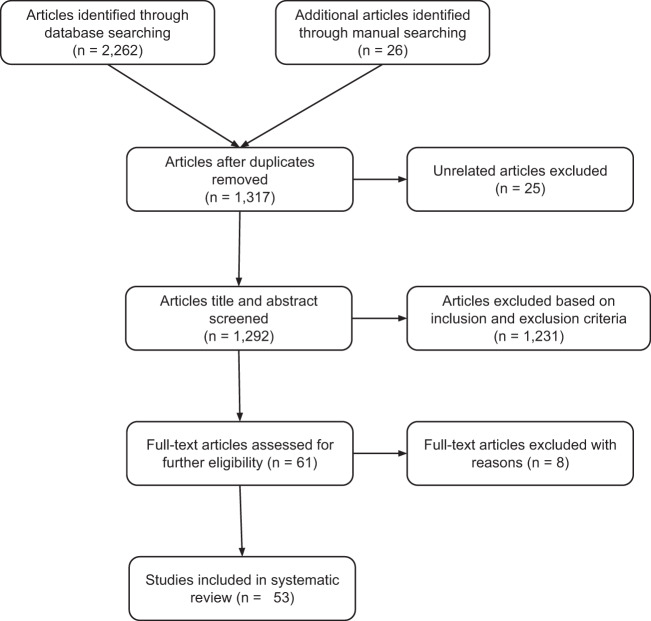
PRISMA flow diagram illustrating the screening and selection of literature.

In total, 2262 articles were identified through database searching and an additional 26 articles were obtained through manual searching of cross-references and tables of content of relevant journals. After removing duplicate and unrelated articles, the titles and abstracts of 1292 articles were screened. The evaluation of titles and abstracts resulted in the exclusion of 1231 articles. In total, 61 articles were selected for full-text review, and eight articles^44–51^ were excluded due to unclear outcomes, mixed diagnosis of the study population and use of a non-statistical method of clustering or clustering based on different phenotypes of schizophrenia. Finally, data were extracted from 53 longitudinal and cross-sectional studies. The PRISMA flow diagram of screening and the selection process is shown in Fig. 1.

### Overview of included studies

The included 53 studies were conducted globally in 30 countries and published over a decade from 2009 to 2020. Seventeen studies were conducted in the USA and few studies were internationally conducted. Of these, 19 studies were longitudinal that involved 11,684 patients, 1059 siblings and 2194 controls or general population from more than eight countries, whereas 34 studies were cross-sectional that involved 6138 patients, 7670 siblings, and 3326 controls from 14 countries. Most of the longitudinal studies examined trajectories of positive and negative symptoms in patients, whereas most of the cross-sectional studies explored cognitive subtypes in patients. Only one longitudinal study^52^ and three cross-sectional studies^53–55^ examined cognitive subtypes among siblings. Overall, two to six subtypes of positive and negative symptoms and cognitive deficits were identified.

### Longitudinal studies

In total, 19 longitudinal studies were reviewed that included all population age groups with the duration of follow-up ranged from six weeks to 10 years. The sample size ranged from 138 to 1990 subjects. Even though all studies had a similar aim, they have used slightly different models of trajectory analysis and model selection criteria. Growth mixture modelling (GMM)^17,56,57^, latent class growth analysis (LCGA)^16,19,20,58–61^, mixed-mode latent class regression modelling^22,62,63^, group-based trajectory modelling (GBTM)^52,64–66^ and Ward’s method^67^ were reported data-driven methods. Akaike’s Information Criterion (AIC), Bayesian information criterion (BIC) (i.e., used in most studies), deviance information criterion (DIC), logged Bayes factor, sample size adjusted BIC (aBIC), bootstrap likelihood ratio test [BLRT], Gap statistic, Lo–Mendell–Rubin Likelihood Ratio Test (LMR-LRT) and entropy were reported model selection indices.

Most longitudinal studies, Table 1, investigated the trajectory of positive, negative or both symptoms in patients whereas one study^68^ explored the trajectory of schizotypy in a nonclinical population. Another study^57^ examined the association between positive and negative symptom trajectories in patients. Moreover, three studies examined the long-term trajectories of cognitive impairment in patients, their unaffected siblings and healthy controls^16,52,66^. One study^52^ investigated the association between patients’ and siblings’ cognitive trajectories as well. Overall, these studies characterized the general pattern of identified trajectories as progressive deterioration, relapsing, progressive amelioration and stable, and the detail results are presented per symptom domains as follows.

**Table 1:** Detailed characteristics of longitudinal studies (*n* = 19).

Authors’ and publication year	Country	Research centre/Cohort	Participants	Assessment tool	Frequency of assessment	Duration of follow-up	Method of calculating test score	Method of trajectory analysis	Number, label and distribution (*n*/%) of trajectories	Significant predictors of trajectories^a^
*Positive symptoms*										
Austin 2015^19^	Denmark	Centre for psychiatric research/OPUS trial trail	496 patients with first-episode SSD and <3 months of treatment	SAPS	Five times	10 years	Composite score using global scores	Latent class analysis	Five: response (233/47), delayed response (60/12), relapse (75/15), non-response (64/13) and episodic response (64/13)	Duration of untreated psychosis, global functioning, diagnosis and substance abuse
Pelayo-Terán et al. 2014^65^	Spain	University Hospital Marqués de Valdecilla/Clinical Programme on First‐Episode Psychosis of Cantabria (PAFIP)	161 patients with a first episode of non-affective psychosis and no prior treatment	SAPS	Six times	6 weeks	Sum score	Group-based trajectory modelling	Five: responders (36/22.4), dramatic responders (25/15.2), partial responders (58/36.2), slow partial responders (29/17.9), and non-responders (13/8.3)	Duration of untreated psychosis and cannabis use
Chen 2013^57^	USA	Mulitcenter trial study, mental health outpatient clinics	400 patients with SSD and treated with first- and second-generation antipsychotics	PANSS	Seven times	1 year	Sum score	Growth mixture modelling	Three: Class 1 (41/10), Class 2 (317/79) and Class 3 (43/11)	Positive and negative symptoms
Abdin 2017^20^	Singapore	Institute of Mental Health/Early Psychosis Intervention Programme (EPIP) clinical database.	1724 patients with first-episode psychotic disorder and with no prior or treatment <3 months	PANSS	Five times	2 years	Not clearly reported	Latent class growth analysis	Two: early response and stable trajectory (/87.7), and delayed response (/12.3)	Gender, educational status, duration of untreated psychosis, diagnosis
*Negative symptoms*										
Pelayo-Terán et al. 2014^65^	Spain	University Hospital Marqués de Valdecilla/Clinical Programme on First‐Episode Psychosis of Cantabria (PAFIP)	161 patients with a first episode of non-affective psychosis and no prior treatment	SANS	Six times	6 weeks	Sum score	Group-based trajectory modelling	Five: responders (22/18.8), mild non-responders (44/37.3), moderate non-responders (22/18.3), partial responders (13/11) and poor responders (17/14.5)	Schizophrenia diagnosis
Abdin 2017^20^	Singapore	Institute of Mental Health/Early Psychosis Intervention Programme (EPIP) clinical database.	1724 patients with first-episode psychotic disorder and with no prior or minimal treatment (<12 weeks)	PANSS	Five times	2 years	Not clearly reported	Latent class growth analysis	Four: early response and stable trajectory (/84), early response and relapse trajectory (/5.9), slower response and no response trajectory (/8.9) and delayed response (/1.2)	Occupational status, educational status, diagnosis
Stiekema et al. 2017^64^	Netherlands	Genetic Risk and Outcome of Psychosis (GROUP)	1067 patients with nonaffective psychosis	PANSS (social amotivation)	Three times	6 years	Sum score	Group-based trajectory modelling	Four: low (670/58.0), decreased low (120/14.6), increased (223/21.2), and decreased high (54/6.2)	Age, gender, educational status, ethnicity, marital status, functioning, quality of life, diagnosis, antipsychotics dosage, neurocognitive performance, negative and psosive symptoms
Stiekema et al. 2017^64^	Netherlands	Genetic Risk and Outcome of Psychosis (GROUP)	1067 patients with nonaffective psychosis	PANSS (expressive deficits)	Three times	6 years	Sum score	Group-based trajectory modelling	Four: low (715/63.6), decreased (180/16.6), increased (114/13.9) and high (58/5.9)	Age, gender, educational status, ethnicity, marital status, functioning, quality of life, diagnosis, antipsychotics dosage, neurocognitive performance, negative and psosive symptoms
Gee 2016^61^	UK	National EDEN study	1006 patients with first episode psychosis and receiving treatment for 12 months	PANSS	Three times	1 year	Mean score	Latent class growth analysis	Four: minimal decreasing (674/63.9), mild stable (108/13.5), high decreasing (174/17.1) and high stable (50/5.4)	Gender, family history of non-affective psychosis, poor premorbid adjustment and depression
Austin 2015^19^	Denmark	Centre for psychiatric research/OPUS trial trail	496 patients with first-episode SSD and had received <12 weeks of treatment	SANS	Five times	10 years	Composite score using global scores	Latent class analysis	Four: response (139/28), delayed response (94/19), relapse (129/26) and non-response (134/27)	Gender, social and global functioning, treatment, disorganized symptoms and diagnosis
Chen 2013^57^	USA	Mulitcenter trial study, mental health outpatient clinics	400 patients with SSD and treated with antipsychotics	PANSS	Seven times	1 year	Sum score	Growth mixture modelling	Four: Class 1 (44/11), Class 2 (284/71), Class 3 (9/2), and Class 4 (63/16)	Positive and negative symptoms
Chan et al. 2020^67^	Hong Kong, China	Public mental health service centres	209 patients with first-episode schizophrenia-spectrum disorders	CGI-neg	64 times	10 years	Mean score	Ward’s method	Three: low (117/56.0), improving (61/29.2) and relapsed (31/14.8)	Gender, hospitalization, low educational status, unemployment, duration of untreated psychosis, negative symptoms
Chang et al. 2018^58^	Hong Kong, China	Public psychiatric units	138 patients with first-episode nonaffective psychosis and not received treatment >1 week	HEN	Four times	3 years	Sum score	Latent class growth analysis	Three: minimal-stable (81/59.6), mild-stable (40/29.4) and high-increasing (15/11.0)	Gender, educational status, premorbid adjustment, cognitive performance, depressive symptoms, positive and negative symptoms
*Positive and negative symptoms (PANSS total score)*										
Schennach et al. 2012^60^	German	Multi-centre study/ German Research Network on Schizophrenia (GRNS)	399 patients with schizophrenia spectrum disorder	PANSS	More than 10 times	>5 months	Sum score	Latent class growth analysis	Five: early and considerable response (61/15), rapid and dramatic response (54/14), early and satisfying response (137/34), gradual response (89/22) and partial response (58/15)	Depressive symptoms at admission, functioning, duration of illness, previous hospitalizations, positive and negative symptoms
Stauffer et al. 2011^56^	USA and other countries	Multicentre study	1990 patients with chronic schizophrenia and receiving treatment	PANSS	11 times	≤6 months	Sum score	Growth mixture modelling	Five: dramatic responders (47/2.4), partial responders (1802/90.6), partial responders-unsustained (late) (32/1.6), partial responders-unsustained (early) (28/1.4) and delayed Responders (81/4.1)	Age, gender, ethnicity, weight, age of onset, depression symptoms, extrapyramidal symptoms, aripiprazole treatment
Levine 2010a^22^	12 countries	International cohort/ Johnson & Johnson Pharmaceutical Research and Development	491 patients with early episode psychosis and receiving treatment for >3 months	PANSS	Six times	6 months	Sum score	Mixed-mode latent class regression modelling	Five: stable 1 (91/18.3), stable 2 (104/20.9), stable 3 (132/26.6), improved and stable (76/15.3), and marked improvement) (94/18.9)	Diagnosis of schizophrenia, age of onset, cognitive functioning, premorbid functioning
Levine 2010b^62^	12 countries	International cohort/ Johnson & Johnson Pharmaceutical Research and Development	263 patients with early episode psychosis and receiving treatment for >3 months	PANSS	More than six times	2 years	Sum score	Mixed-mode latent class regression modelling	Five: Trajectory 1 (55/21.0), Trajectory 2 (60/22.9), Trajectory 3 (64/24.4), Trajectory 4 (40/15.2) and Trajectory 5 (44/16.6)	Diagnosis, premorbid functioning, cognitive performance, positive and negative symptoms
Case et al. 2011^17^	3 countries	64 research centres	628 patients with psychosis and treated with antipsychotics	PANSS	Eight times	3 months	Sum score	Growth-mixture modelling	Four: moderate-gradual (420/80.6), rapid (65/12.5), high-gradual (24/4.6), unsustained (12/2.3) improvement	Extrapyramidal and depression symptoms, quality of life, age at onset of illness, ethnicity, positive and negative symptoms, general psychopathology
Chen 2013^57^	USA	Mulitcenter trial study, mental health outpatient clinics	400 patients with SSD and treated with first- and second-generation antipsychotics	PANSS	Seven times	1 year	Sum score	Growth mixture modelling	Three: dramatic and sustained early improvement (70/18), mild and sustained improvement (237/59), and no improvement (82/21)	Positive and negative symptoms
Levine et al. 2012^63^	USA	57 clinical sites	1124 patients with chronic schizophrenia and receiving treatment	PANSS	Eight times	1.5 years	Sum score adjusted for the baseline score	Mixed-mode latent regression modelling	Three: low deteriorators (778/69.2), responders (212/18.9) and high deteriorators (134/11.9)	Type of antipsychotics, exacerbation, positive and negative symptoms
Jager 2014^59^	Germany	ELAN study, psychiatric hospitals	268 patients with SSD and receiving treatment for >1 year	PANSS	Five times	2 years	Sum score	Latent class growth analysis	Two: amelioration/decrease in all symptoms (154/60 and stable positive/negative symptoms and deteriorating general psychopathology symptoms (103/40)	Global functioning, gender, age, living situation and involuntary admission
*Cognitive deficits*										
Habtewold et al. 2020^66^	Netherlands	Genetic Risk and Outcome of Psychosis (GROUP)	1119 patients with nonaffective psychosis, 1059 siblings, and 586 controls	NTB	Three times	6 years	PCA, sum of component scores	Group-based trajectory modelling	Six: very severe (199/0.8), severe (159/6.2), moderate (384/15.1), mild (684/25.8), normal (1056/33.5), and high (462/18.5)	Polygenic risk score of schizophrenia
Islam et al. 2018^52^	Netherlands	Genetic Risk and Outcome of Psychosis (GROUP)	1119 patients with nonaffective psychosis, 1059 siblings, and 586 controls (results are only for patients)	NTB	Three times	6 years	Gender and age adjusted z-score and then averaging	Group-based trajectory modelling	Five: severely altered (109/10.7), moderately altered (312/28.4), mildly altered (377/30.4), normal (290/26.7), and high (31/3.8) performer	Education, IQ, premorbid functioning, and positive and negative symptoms
Islam et al. 2018^52^	Netherlands	Genetic Risk and Outcome of Psychosis (GROUP)	1119 patients with nonaffective psychosis, 1059 siblings, and 586 controls (results are only for siblings)	NTB	Three times	6 years	Gender and age adjusted z-score and then averaging	Group-based trajectory modelling	Four: moderately altered (132/13.0), mildly altered (260/25.1), normal performer (413/37.6), and high performer (254/24.2)	Age, gender, education, ethnicity, IQ, premorbid functioning, positive symptoms, frequency of psychotic experiences, and neurocognitive performances
Thomspson et al. 2013^16^	USA	University of California, San Diego Advanced Centre in Innovation in Services and Interventions Research (ACISIR)	201 old clinically stable outpatients with schizophrenia and 67 controls	MDRS	Four times	3.5 years	Sum score	Latent growth curve model	Three: high and stable (101/50), low and modestly declining (81/42), low and rapidly declining (19/10)	Negative symptoms, living situation, years of education, global cognition
*Schizotypy*										
Wang et al. 2018^68^	China	University of Chinese Academy of Sciences/Key Laboratory of Mental Health	1541 college students	CPPS (4 subscales)	Four times	1.5 years	Sum score	Latent class growth analysis	Four: non-schizotypy (1113/72.2), stable-high schizotypy (73/4.74), high-reactive schizotypy (142/13.8), low-reactive schizotypy (213/13.8)	Male gender, severe schizotypy

*CGI-neg* Clinical Global Impressions-Schizophrenia scale for negative symptoms, *CPPS* Chapman Psychosis Proneness Scales, *HEN* High Royds Evaluation of Negativity Scale, *MDRS* Mattis Dementia Rating Scale, *NTB* Neuropsychological Test Battery (seven tests were used), *PANSS* Positive and Negative Syndrome Scale, *SANS* Scale for the Assessment of Negative Symptoms, *SAPS* Scale for the Assessment of Positive Symptoms, *SSD* Schizophrenia spectrum disorder.

^a^Results from pairwise comparisons, univariable or multivariable logistic regression analyses.

#### Positive symptoms

**Fig. 2:**
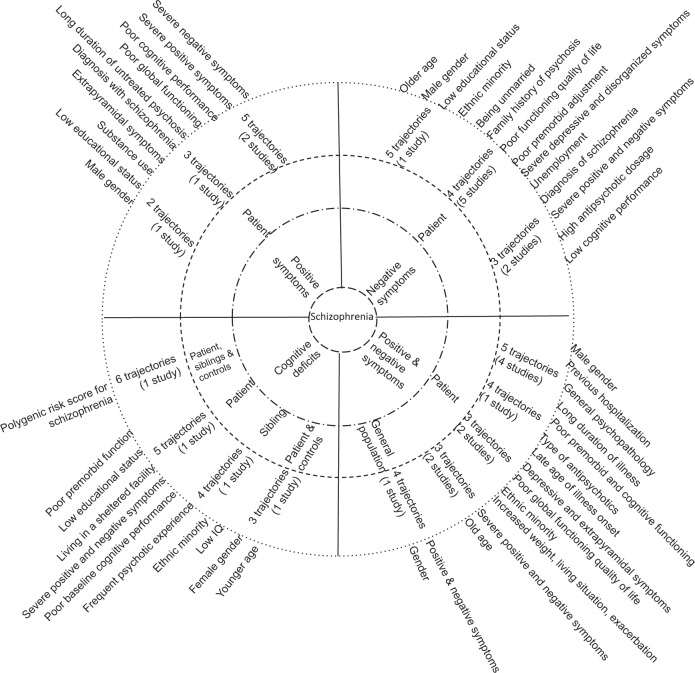
Schizophrenia spectrum circle illustrating the schizophrenia symptoms and cognitive deficits (innermost circle), sample groups (inner circle), identified trajectories (outer circle) and predictors (outermost circle) in longitudinal studies. Findings are read and interpreted based on the line up in the circle.

As presented in Table 1a, four studies^19,20,57,65^ investigated the trajectory of positive symptoms in patients with first-episode schizophrenia spectrum disorders with no or prior antipsychotics treatment for less than three months. The duration of follow-up and frequency of assessment ranged from six weeks to 10 years and five to seven times, respectively. Two studies^19,65^ have used the Scale for the Assessment of Positive Symptoms (SAPS) to assess positive symptoms and identified five trajectories with more than one-third of patients subtyped as decrease positive symptoms or good responders. The other two studies used the Positive and Negative Syndrome Scale (PANSS) tool to assess positive symptoms and identified three trajectories that most of them grouped to class two^57^ and two trajectories being in the most of the cases early response and stable trajectory over time^20^. The identified predictors were male gender, low educational status, substance use, diagnosis with schizophrenia, long duration of untreated psychosis, poor global functioning, and severe baseline positive and negative symptoms (Fig. 2).

#### Negative symptoms

Eight longitudinal studies^19,20,57,58,61,64,65,67^ explored negative symptom trajectories among patients with first-episode non-affective psychosis with no prior or minimal treatment up to three months (Table 1b). Two studies^19,65^ used the Scale for the Assessment of Negative Symptoms (SANS), four studies^20,57,61,64^ used the PANSS scale and two studies used the High Royds Evaluation of Negativity Scale^58^ and Clinical Global Impressions-Schizophrenia scale^67^ to assess negative symptoms. The duration of follow-up and frequency of assessment ranged from 6 weeks to 10 years and three to 64 times, respectively. Five studies^19,20,57,61,64^ identified four trajectories of negative symptoms with variable patterns, whereas one study^65^ found five trajectories with approximately half of them had persistent symptoms or poor response to treatment. The other two studies^58,67^ found three trajectories with most of the participants had minimally stable negative symptoms. Our review depicted that trajectories of negative symptoms were predicted by older age, male gender, low educational status, ethnic minority, being unmarried, family history of psychosis, long duration of untreated psychosis, poor premorbid adjustment, severe depressive and disorganized symptoms, diagnosis of schizophrenia, unemployment, poor functioning and quality of life, high antipsychotics dosage, low cognitive performance, and high level of baseline negative and positive symptoms (Fig. 2).

#### Positive and negative symptoms

Combining both positive and negative symptom dimensions, which is illustrated in Table 1c, eight studies^17,22,56,57,59,60,62,63^ inspected trajectories in patients with first-episode or chronic schizophrenia with antipsychotics treatment for more than three months and all of these studies used the PANSS scale to measure positive and negative symptoms. The duration of follow-up and frequency of assessment ranged from three months to two years and five to ten times, respectively. Among these studies, four^22,56,60,62^ of them revealed five trajectories, two^57,63^ of them revealed three trajectories, one study^17^ found four trajectories and another study^59^ found two trajectories with substantial difference in the nature, pattern and distribution of trajectories. Symptom trajectories were predicted by older age, male gender, ethnic minority, increased weight, diagnosis with schizophrenia, late age of illness onset, depressive and extrapyramidal symptoms, general psychopathology, type of antipsychotics treatment (e.g., aripiprazole, olanzapine), exacerbation, long duration of illness, poor premorbid and cognitive functioning, low global functioning and quality of life, living situation, involuntary admission, previous hospitalization and severe baseline positive and negative symptoms (Fig. 2).

#### Cognitive deficits

As shown in Table 1d, three studies investigated the trajectories of global cognitive deficits in patients with first-episode psychosis patients, their siblings and healthy controls^52,66^, and clinically stable outpatients with schizophrenia (SCZ) together with healthy controls^16^. The first six-year longitudinal study^52^, which cognitive function was assessed by the cognitive battery test, depicted five trajectories of cognitive impairment in patients (i.e., most of them with mild to moderate deficits) and four trajectories in healthy siblings (i.e., most of them had normal cognitive function). The second study^66^, which was the follow-up of the previous study, found six cognitive trajectories (i.e., nearly half of the population had mild to severe cognitive impairment) by combining patients, siblings and controls. The third longitudinal study^16^ have used the Mattis Dementia Rating Scale and reported three trajectories (i.e., half of them with high and stable trajectory) of global cognitive function by combining patients and controls. Two studies found that patients with poor cognitive trajectories had younger age, low educational status, non-Caucasian ethnicity, lived in a sheltered facility, low IQ, poor premorbid adjustment, severe positive and negative symptoms, and low baseline cognitive performance^16,52^. Likewise, siblings with poor cognitive trajectories had younger age, female gender, low educational status, non-Caucasian ethnicity, low IQ, poor premorbid adjustment, severe schizotypy, frequent positive psychotic experience, and low baseline cognitive performance (Fig. 2)^52^. One study discovered that polygenic risk score for schizophrenia significantly predicted poor long-term cognitive trajectory in combined samples of patients, siblings and controls^66^.

#### Schizotypy

A single longitudinal study assessed schizotypy in healthy college students using the Chapman Psychosis Proneness Scales (CPPS) and found four trajectories, in which nearly three-fourths of students were categorized as non-schizotypal^68^. This study also found that male gender and a high level of baseline schizotypy significantly predicted trajectories (Table 1e, Fig. 2).

In summary, when we inspecting the longitudinal study’s findings shown in Table 1, studies that found the same number of trajectories were substantially different concerning participants composition (patient, sibling and controls), assessment instruments, symptom dimensions, frequency of assessment, duration of follow-up, methods used to generate a composite score, data-driven methods applied, label, proportion, pattern and type of trajectories, and identified predictors. In addition, there was no link between the numbers and types of trajectories and the use of trajectory analysis methods, study population and symptom dimensions.

### Cross-sectional studies

Of the 53 included studies, 34 studies were cross-sectional (Table 2) that conducted in different groups of population. The total sample size per study ranged from 62 to 8231 individuals irrespective of participants’ diagnostic status. The reported clustering methods were K-means or non-hierarchical clustering analysis^21,53,55,69–76^, Ward’s method or hierarchical analysis^77–83^, K-means clustering and Ward’s method^18,38,54,84–89^, latent class or profile analysis^15,90,91^ and two-step cluster analysis^92–94^. One study^95^ identified clusters using a combination of clinical/empirical and statistical clustering methods. The model selection criteria or similarity metrics were visual inspections of the dendrogram, Pearson correlation, squared Euclidean distance (i.e., the most common index), agglomeration coefficients, Dunn index, Silhouette width, Duda and Hart index, elbow test, variance explained, inverse scree plot, average proportion of non-overlap, AIC, BIC, aBIC, Schwarz’s BIC, Lo–Mendell–Rubin (LMR) test, adjusted LMR and BLRT.

**Table 2:** Detailed characteristics of cross-sectional studies (*n* = 34).

Authors’ and publication year	Country	Research centre/Cohort	Participants	Assessment tool	Method of calculating score	Method of clustering	Number, label and distribution of clusters (*n*/%)	Significant correlates of clusters^a^
*Positive symptoms*								
Chang 2015^83^	Korea	Seoul National University Hospital and Boramae Medical Center	111 patients with schizophrenia	LSHS-R	Sum score	Ward’s cluster analysis	Three: perception dimension and perception-cognition dimension (cluster 2 and 3)	Not reported.
*Negative symptoms*								
Strauss et al. 2013^85^	USA	Veterans Affairs Greater Los Angeles Healthcare System	199 patients with schizophrenia	SANS	Mean factor scores (PCA)	Ward’s and K-means cluster analysis	Three: diminished expression (41/20.6), avolition–apathy (85/42.7) and low negative symptoms (75/37.7)	General psychopathology, severity of positive and negative symptoms, social anhedonia, attitude, global functioning, social cognition, hospitalization
Ahmed 2018^15^	USA	Maryland Psychiatric Research Center (MPRC)	706 patients with chronic schizophrenia	SDS	Sum score	Latent class analysis with prior hypothesis	Three: deficit (128/19.3), persistent (174/25.1) and transient (404/55.6)	Sex, season of birth, ethnicity, years of education, illness onset, positive symptoms, neurocognitive performance, premorbid adjustment, psychosocial functioning
*Positive and negative symptoms*								
Trauelsen et al. 2016^69^	Denmark	OPUS	97 patients with first-episode non-affective psychosis and 101 controls	PANSS	Z-scores	K-means cluster analysis	Four: low positive and negative symptoms (39/40.2), high positive and low negative (15/15.5), low positive and high negative (16/16.5), and high positive and high negative (24/24.7)	Metacognition
Talpalaru et al. 2019^77^	Multinational	North-western University Schizophrenia Data and Software Tool (NUSDAST) dataset	104 patients with schizophrenia and 63 healthy controls	SAPS, SANS	Z-scores	Ward’s cluster analysis	Three: high positive and negative symptom (27/26.0), predominantly positive symptom (36/34.6), and low symptom (41/39.4)	Gender
Craddock 2018^21^	USA	National Institute of Mental Health (NIMH)/Childhood-onset schizophrenia (COS) cohort	125 patients with childhood-onset schizophrenia (COS)	SAPS, SANS	Factor score (CFA)	K-means cluster analysis	Three: low positive and negative (37/29.6), high negative low positive (33/26.4), and high positive and negative (55/44.0)	IQ, global functioning, positive and negative symptoms
*Cognitive deficits*								
Dawes 2011^88^	USA	University of California/San Diego (UCSD) Advanced Center for Innovation in Services and Interventions Research (ACISIR)	144 patients with schizophrenia or schizoaffective disorder	Comprehensive neuropsychological test battery (7 tests)	Sum of deviation scores adjusted to age, gender, education and ethnicity	Ward’s and K-means cluster analysis	Five: low visual learning and memory (19/13.2), low auditory and visual learning, memory and abstraction/cognitive flexibility (38/26.4), low abstraction/cognitive flexibility (40/27.8), low auditory learning, memory and abstraction/cognitive flexibility (17/11.8), and low visual learning, memory and abstraction/cognitive flexibility (30/20.8)	Educational status, ethnicity
Lewandowski 2018^87^	USA	McLean Hospital/Schizophrenia and Bipolar Disorder Program (SBDP)	120 patients with psychosis and 31 healthy controls	MCCB (10 subtests)	Age and gender adjusted T-scores	Ward’s and K-means cluster analysis	Four: normal (39/32.5), mildly impaired (42/35.0), moderately impaired (18/15.0) and significantly impaired (21/17.5)	Educational status, premorbid IQ, state mania, positive and negative symptoms, antipsychotic dosage, cognition, community functioning
Reser et al. 2015^86^	Australia	Early Psychosis Prevention and Intervention Centre (EPPIC)	128 patients with a first-episode psychosis	Comprehensive cognitive battery test (15 tests)	Range standardized test scores	Ward’s and K-means cluster analysis	Four: poor visual recognition memory (26/20.3), flat profile (46/35.9), strong performance (25/19.5) and poor performance (31/24.2)	Age, IQ (premorbid and current), years of education, negative symptoms, neurocognitive performance
Geisler 2015^75^	USA	Four research centers (MGH, UI, UMN, UNM)/Mind Clinical Imaging Consortium (MCIC) study of schizophrenia	129 patients with schizophrenia and 165 healthy controls	Comprehensive neuropsychological test battery (18 tests)	PC score (PCA)	K-means cluster analysis	Four: diminished verbal fluency (38/29.4), diminished verbal memory and poor motor control (26/20.2), diminished face memory and slowed processing (21/16.3), and diminished intellectual function (44/34.1)	Duration of illness, positive symptoms, years of education, premorbid adjustment, cortical thickness, neural activity
Rangel et al. 2015^91^	Colombia	Universities of Antioquia, Pontificia Bolivariana, Nacional of Colombia	253 patients with schizophrenia	Neuropsychological tests (5 tests)	Not reported	Latent classes analysis	Four: global cognitive deficit (74/29.2), memory and executive function deficit (75/29.6), memory and facial emotion recognition deficit (60/23.7), and without cognitive deficit (44/17.4)	Gender, age, negative symptoms, global functioning, employment status, adherence to treatment, neurocognitive performance, depression
Lewandowski 2014^18^	USA	McLean Hospital/Schizophrenia and Bipolar Disorder Program (SBDP)	167 patients with psychosis	Neuropsychological battery test (5 tests)	Z-scores adjusted to age or age and education	Ward’s and K-means cluster analysis	Four: globally normal (46/27.5), normal processing speed/executive function (42/25.1), normal visuospatial function (35/21.0) and globally impaired (44/26.3)	Cognition, age, educational attainment, antipsychotics dosage, positive and negative symptoms, community functioning
Dickinson et al. 2019^92^	USA	National Institute of Mental Health Clinical Center	540 schizophrenia patients, 247 unaffected siblings, and 844 control subjects	WRAT, WAIS IQ	Average of z-scores (based on controls mean and SD)	Two-step Cluster analysis	Three: cognitively stable (198/37), preadolescent impairment (105/19) and adolescent decline (237/44)	Polygenic risk scores (schizophrenia, cognition, education, ADHD), educational status, employment, positive and negative symptoms, global functioning, cognitive performance
Smucny et al. 2019^90^	USA	CNTRACS consortium	223 psychosis patients and 73 healthy controls	Neuropsychological tests (3 tests)	Z-score and Factor score	Latent profile analysis (LPA)	Three: low (15/6.7), moderate (66/29.6) and high (142/63.7)	Negative, positive, disorganization, mania, and depressed mood symptoms, functioning, educational status, neurocognitive perfomance
Crouse et al. 2018^81^	Australia	Brain and Mind Research Institute	135 patients with a psychosis-spectrum illness and 50 healthy controls	CANTAB (9 tests)	Age-adjusted Z-scores	Ward’s cluster analysis	Three: normal-range (46/34.0), mixed (58/43.0) and grossly impaired (31/23.0)	Socio-occupational functioning, neurocognitive performance, gender, diagnosis, risky drinking, employment status, educational status, premorbid IQ, negative symptoms
Sauve et al. 2018^38^	Canada	Douglas Mental Health University Institute (DMHUI)/ PEPP-Montreal program	201 patients with psychosis on treatment and 125 healthy controls	CogState Schizophrenia Battery (13 tests)	Composite scores standardized to controls	Ward’s and K-means cluster analyses	Three: no impairment (169/51.8), generally impaired (39/12.0) and intermediately impaired (118/36.2)	IQ, severity of positive symptoms, age, years of education, stage of illness, antipsychotics dosage
Bechi 2018^93^	Italy	IRCCS San Raffael Scientific Institute	452 patients with stable schizophrenia	BACS, WAIS-R	Mean score adjusted to age and education	Two-step cluster analysis (both scores together)	Three: high (135/29.9), medium (173/38.3) and low (144/31.8) (for all sample)	Age, years of education, age of onset, negative and positive symptoms, IQ, cognition
Uren et al. 2017^84^	Australia	Early Psychosis Prevention and Intervention Centre (EPPIC)	133 patients with first episode psychosis and 46 controls	Comprehensive battery test (14 tests)	Z-scores	Ward’s and K-means cluster analysis	Three: severe global impairment (24/13.4), moderate impairment (73/40.8) and intact (82/45.8)	Age, premorbid IQ, positive and negative symptoms, cognitive performance, years of education, functioning
Ohi et al. 2017^53^	Japan	Kanazawa Medical University Hospital/ Kanazawa Medical University	81 patients with schizophrenia, 20 relatives and 25 healthy controls	BACS (6 subscales)	Age- and gender-corrected raw scores	K-means cluster analysis	Three: neuropsychologically normal (36/28.6), intermediately impaired (60/47.6) and globally impaired (30/23.8)	Clinical diagnosis, neurocognitive performance, years of education, premorbid IQ, antipsychotics dosage
Prouteau et al. 2017^80^	France	Public psychiatric hospitals	69 patients with schizophrenia spectrum disorders	Objective: Neuropsychological tests (6 tests) Subjective: SSTICS	Standardized Z-scores	Ward’s cluster analysis	Three: high cognitive impairment/moderate cognitive complaints (26/37.7), good cognitive functioning/moderate cognitive complaints (22/31.9) and moderate cognitive impairment/high cognitive complaints (21/30.4)	Age, educational status, negative symptoms, quality of life, anxiety, depression, stigma, neurocognitive performance
Rodrigez et al. 2017^79^	Czech	National Institute of Mental Health	28 patients with first-episode SSD and 91 healthy controls	Neuropsychological battery tests (15 tests)	Z-scores standardized using controls	Ward’s cluster analysis	Three: generalized severe (10/35.7), partial mild (7/25.0) and near normal (11/39.3)	Neurocognitive performance
Rocca et al. 2016^94^	Italy	Multicentre study/Italian Network for Research on Psychoses (NIRP)	809 patients with schizophrenia and 780 controls	MCCB (3 tests)	Z-scores of scales	Two-step cluster analysis	Three: unimpaired (340/42), impaired (408/50.4) and very impaired (61/7.5)	Age, educational status, cognitive performance, functioning, positive and negative symptoms, disorganization
Wells et al. 2015^95^	Australia	Australian Schizophrenia Research Bank (ASRB)	534 patients with schizophrenia or schizoaffective disorder and 635 healthy controls	Neuropsychological tests (5 tests)	Z-scores standardized by healthy controls	Ward’s and K-means cluster analysis, and clinical method	Three: preserved (157/29), deteriorated (239/44) and compromised (138/26)	Age, years of education, age onset of illness, gender, neurocognitive performance, positive and negative symptoms, functioning
Gilbert 2014^82^	Canada	Institut en santé mentale de Québec	112 patients with schizophrenia	Cognitive battery test (> 8 tests)	Average Z-scores	Ward’s cluster analysis	Three: generally impaired (18/16.1), selectively impaired (46/41.1) and near normal (48/42.8)	IQ, gender, socioeconomic status, cognition, antipsychotics dosage, global functioning, positive and negative symptoms
Quee et al. 2014^54^	Netherlands	Genetic Risk and Outcome of Psychosis (GROUP)	654 health siblings of patients with schizophrenia	Neuropsychological battery test (8 tests)	Mean score of gender and age-adjusted z-scores	Ward’s and K-means cluster analysis	Three: normal (192/29.4), mixed (228/34.8) and impaired (234/35.8)	Age, educational status, IQ, premorbid adjustment, positive schizotypy
Ochoa et al. 2013^71^	Spain	Hospital and community psychiatric services	62 patients with a first-episode psychosis	Neuropsychological battery tests (5 tests)	Demographically adjusted score	K-means cluster analysis	Three: higher neurodevelopment contribution (14/22.6), higher genetic contribution (30/48.4) and lower neurodevelopment contribution (18/29.0)	Neurocognition performance, premorbid IQ, neurological soft signs, premorbid adjustment, family history of mental disorders, obstetric complications
Bell 2010^76^	USA	Community mental health center (CMHC)	151 patients with schizophrenia spectrum disorder (stable)	HVLT-R	Sum score	K-means cluster analysis (with prior hypothesis)	Three: nearly normal (52/34.4), subcortical (68/45.0) and cortical (31/20.5)	Educational status, neurocognitive performance, social cognition
Potter et al. 2010^70^	USA	University of Massachusetts	73 patients with schizophrenia and 74 controls	Neuropsychological tests (6 tests)	Scaled scores	K-means cluster analysis	Three: intellectually compromised (31/42), intellectually deteriorated 21(/29) and intellectually preserved (21/29)	Negative symptoms, neurocognitive performance, educational status, general psychopathology
Wu et al. 2010^78^	Taiwan	Psychiatric rehabilitation hospital	76 patients with schizophrenia	BNCE (10 subscales)	Mean scores	Ward’s cluster analysis	Three: near normal (34/45), deteriorated conceptual thinking (20/26), and anomia and impaired executive function (22/29)	Severity of negative symptoms
Bechi 2018^93^	Italy	IRCCS San Raffael Scientific Institute	52 patients with stable schizophrenia	BACS, WAIS-R	Sum score	Two-step cluster analysis (both scores together)	Two: high (30/57.7) and medium (22/42.3) (subsamples with high pre-morbid IQ)	Age, years of education, age of onset, negative and positive symptoms, IQ, cognition
*Negative symptoms and cognitive deficits*								
Lysaker et al. 2009^74^	USA	Roudebush VA Medical Center and Community Mental Health Center (CMHC)	99 patients with stable schizophrenia or schizoaffective disorder and on treatment	PANSS, CPT	Normalized z-scores	K-means cluster analysis	Four: low negative/relatively better attention (31/31.3), low negative/relatively poor attention (20/20.2), high negative/ relatively poor attention (28/28.3), and high negative/relatively better attention (20/20.2)	Self-esteem, attention performance, acceptance of stigma, severity of positive and negative symptoms, social functioning
Bell 2013^89^	USA	Community mental health center (CMHC)	77 outpatients with stable schizophrenia or schizoaffective disorder	SANS, PANSS, MSCEIT	Sum score	Ward’s and K-means cluster analysis	Three: high negative symptom (24/31.2), low negative symptom with higher social cognition (27/35.1), and low negative symptom with poorer social cognition (26/33.7)	Quality of life, hospitalization, marital status, negative symptoms, social cognition
*Schizotypy*								
Lui et al. 2018^55^	China	Castle Peak Hospital	194 unaffected first-degree relatives of patients with schizophrenia	CPPS (4 subscales)	Sum score	K-means cluster analysis	Four: high positive (33/17.0), high negative (66/34.0), mixed (27/13.9) and low (64/32.9) schizotypy	Positive and negative schizotypy, everyday life pleasure experiences, emotional expressivity
Wang et al. 2012^72^	China	Neuropsychology and Applied Cognitive Neuroscience Laboratory	418 healthy college students	CPPS	Normalized component score (PCA)	K-means cluster analysis	Four: low (148/35.4), high positive (71/17.0), high negative (116/27.7), and mixed (high positive and negative) (83/19.9) schizotypy	Psychotic-like symptoms, depression, and social function, emotional expression, pleasure experiences, somatic symptoms, neurocognitive functioning, proneness to positive and negative symptoms
Barrantes-Vidal et al. 2010^73^	USA	University of North Carolina at Greensboro (UNCG)	6,137 healthy college students	CPPS	Normalized component score (PCA)	K-means cluster analysis	Four: low (2,137/35), high positive (1,895/31), high negative (1,352/22), and mixed (high positive and negative) (753/12) schizotypy	Severity of positive and negative schizotypy, gender, social functioning, psychotic-like experiences, depression, substance use and abuse, schizoid and negative symptoms, personality, social adjustment
Chang 2015^83^	Korea	Seoul National University Hospital and Boramae Medical Center	223 nonclinical population	LSHS-R	Sum score	Ward’s cluster analysis	Two: Perception dimension and Cognitive dimension	Not reported.

*BACS* Brief Assessment of Cognition in Schizophrenia, *BNCE* Brief Neuropsychological Cognitive Examination, *CANTAB* Cambridge Neuropsychological Test Automated Battery, *CPPS* Chapman Psychosis Proneness Scales, *CPT* Continuous Performance Tests, *HVLT-R* Hopkins Verbal Learning Test—revised, *LSHS-R* Launay–Slade Hallucination Scale—revised, *MCCB* MATRICS Consensus Cognitive Battery, *MSCEIT* Mayer-Salovey-Caruso Emotional Intelligence Test, *PANSS* Positive and Negative Syndrome Scale, *SANS* Scale for the Assessment of Negative Symptoms, *SAPS* Scale for the Assessment of Positive Symptoms, *SDS* Schedule for the Deficit Syndrome, *SSD* Schizophrenia spectrum disorder, *SSTICS* Subjective Scale to Investigate Cognition in Schizophrenia, *WAIS-R* Wechsler Adult Intelligence Scale—revised, WRAT Wide-Range Achievement Test.

^a^Results from pairwise comparisons, univariable or multivariable logistic regression analyses.

Among the 34 studies (Table 2), 22 studies^18,38,53,54,70,71,75,76,78–82,84,86–88,90–95^ reported cognitive clusters in patients with first-episode, stable or chronic schizophrenia with or without antipsychotics treatment and one study^54^ reported cognitive clusters in unaffected siblings. Other studies investigated trajectories of negative symptoms^15,85^, positive symptoms^83^, positive and negative symptoms^21,69,77^ in patients and positive and negative schizotypy in a nonclinical population^55,72,73,83^. Furthermore, two studies^75,90^ investigate the data-driven clusters by combining cognitive deficit and negative symptoms. Details on clusters and correlates of clusters presented per symptom dimensions as follows.

#### Positive symptoms

Only one study^83^ assessed hallucinatory experience in patients with schizophrenia using Launay–Slade Hallucination Scale-Revised (LSHS-R) and identified three clusters (Table 2a)^83^. Given this was an explanatory study, correlates of clusters were not studied.

#### Negative symptoms

**Fig. 3:**
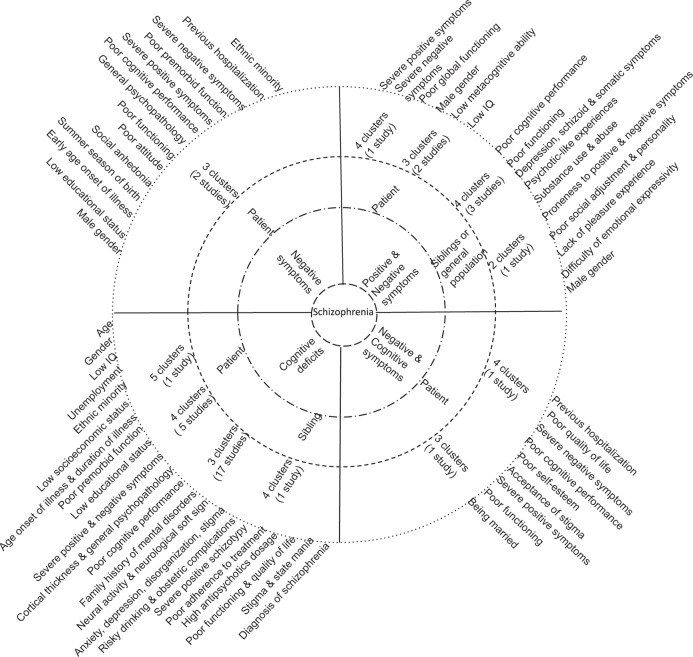
Schizophrenia spectrum circle illustrating the schizophrenia symptoms and cognitive deficits (innermost circle), sample groups (inner circle), identified clusters (outer circle) and correlates (outermost circle) in cross-sectional studies. Findings are read and interpreted based on the line up in the circle.

Two studies^15,85^ reported three clusters of patients with (chronic)schizophrenia based on the negative symptoms that assessed by the SANS scale^85^ and Schedule for the Deficit Syndrome (Table 2b)^15^. Identified clusters were significantly correlated with male gender, ethnic minority, low educational status, summer season of birth, early age onset of illness, severity of positive and negative symptoms, poor cognitive performance, poor functioning, high level of general psychopathology, previous hospitalization, poor premorbid adjustment, social anhedonia and poor attitude (Fig. 3).

#### Positive and negative symptoms

Two studies^21,77^ assessed positive and negative symptoms in patients with childhood-onset or first-episode schizophrenia using the SAPS and SANS scales, respectively and found three clusters, while another study^69^ used the PANSS scale and found four clusters (Table 2c). Reported symptom clusters were characterized as low positive and negative symptoms, high positive and low negative, low positive and high negative, and high positive and high negative though the patterns and distributions of clusters were different across studies. Identified clusters were significantly correlated with male gender, low IQ, poor global functioning, poorer metacognitive ability, and high level of positive and negative symptoms (Fig. 3).

#### Cognitive deficits

Of the 22 studies conducted on neurocognitive deficits, 17 studies^38,53,70,71,76,78–82,84,90,92–95^ found three clusters, five studies^18,75,86,87,91^ reported four clusters and one study^88^ discovered five clusters among patients (Table 2d). Most studies assessed global cognitive function using a comprehensive neuropsychological test that included three to 18 cognitive subtests. Poor cognitive function in patients was associated with age, gender, non-Caucasian ethnicity, low socioeconomic and educational status, poor premorbid adjustment, low premorbid and current IQ, early age of illness onset, long duration of illness, severe positive and negative symptoms, poor social cognition, high antipsychotics dosage, use of second-generation antipsychotics, and poor functioning and poor quality of life (Fig. 3). In siblings, one study^54^ found three cognitive clusters in unaffected siblings that associated with young age, low educational status, low IQ, poor premorbid adjustment and severe positive schizotypy (Table 2d, Fig. 3)^54^. One study^92^ found that polygenic score (PRS) for schizophrenia, cognition, educational attainment and attention deficit hyperactivity disorder (ADHD) correlated with cognitive clusters in patients and their unaffected siblings.

#### Negative symptoms and cognitive deficits

One study^89^ found three clusters of (out)patients with stable schizophrenia spectrum disorder by combining social cognition that assessed by the Mayer-Salovey-Caruso Emotional Intelligence Test and negative symptoms that assessed by the PANSS scale, whereas another study^74^ found four clusters in patients by combining neurocognition that assessed by Continuous Performance Tests and negative symptom that assessed by the PANSS scale (Table 2e). Clusters were significantly correlated with being unmarried, poor self-esteem, low cognitive (attention, social) performance, stigma, severity of positive and negative symptoms, poor social functioning and quality of life, and previous hospitalization (Fig. 3).

#### Schizotypy

Three studies investigated schizotypy in unaffected first-degree relatives of patients with schizophrenia^55^ and healthy college students^72,73^ using the CPPS scale and found four clusters, whereas another study^83^ found two clusters based on hallucinatory experience that assessed by LSHS-R scale in healthy general population (Table 2f). Schizotypy clusters were significantly associated with male gender, lack of pleasure experiences, difficulty of emotional expression, psychotic-like symptoms, severity of positive and negative schizotypy, depressive, schizoid and somatic symptoms, poor social and cognitive functioning, substance abuse and poor personality (Fig. 3).

To summarize, as we observed in longitudinal studies, cross-sectional studies that found the same number of clusters were conducted in a different group of samples and used various assessment instruments and methods of generating composite scores and clustering. The labeling, pattern, proportion, and type of clusters were remarkably different. Generally, three clusters were the most replicated number of clusters and characterized by low (severe deficits), mixed (intermediate deficits) and high (intact or normal performance) cognitive function. In addition, cognitive clustering, such as verbal fluency deficit, verbal memory and executive function deficit, face memory and processing deficits, or global cognitive deficits were revealed. Cross-sectional studies that found the same number of clusters were largely different in the characteristics of study population, pattern of identified clusters, symptom dimensions, methodology of assessment, applied data-driven methods and identified associated factors.

Overall, as shown in Table 3, the reviewed studies reported two to six clusters or trajectories and 58 factors that linked with identified clusters and/or trajectories across all study participants and symptom dimensions. The most common associated factors were old age, male gender, non-Caucasian ethnicity, low educational status, late age of illness onset, diagnosis of schizophrenia, several general psychopathology and depressive symptoms, severe positive and negative symptoms, low cognitive performance, and poor premorbid functioning, quality of life and global functioning.

**Table 3:**
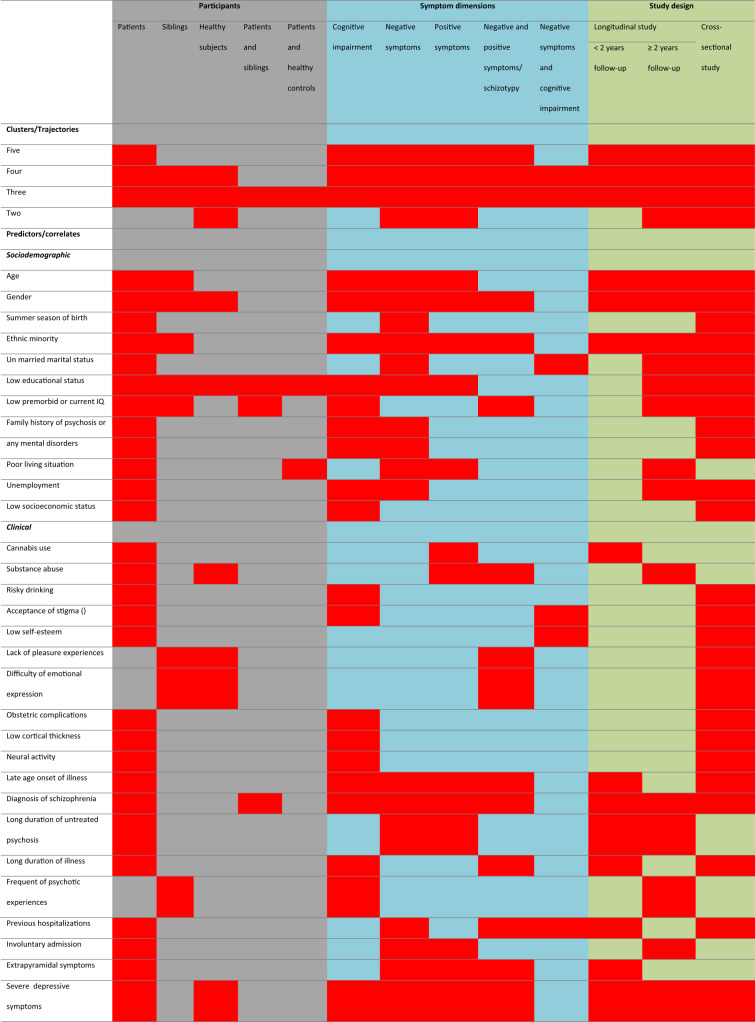

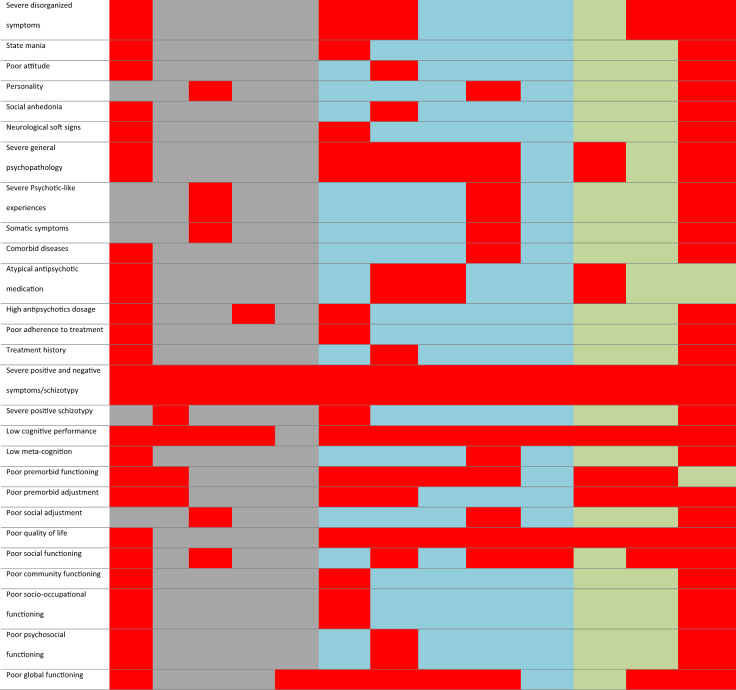
Heatmap summary of clusters/trajectories and predictors across study participants, symptom dimensions and study design.

This table/map can only be read and interpreted horizontally. For example, five clusters/trajectories were found in both longitudinal and cross-sectional studies among patients based on schizophrenia symptoms and cognitive deficits [all red boxes]. The same procedure applies to predictors. For example, age found to be the predictor of clusters/trajectories of schizophrenia symptoms and cognitive deficits in longitudinal and cross-sectional studies among patients and siblings [all red boxes].

## Discussion

To our knowledge, this is the first comprehensive systematic review based on recent cross-sectional and longitudinal data-driven studies in positive and negative symptoms, and cognitive deficits in patients with schizophrenia spectrum disorders, their relatives and healthy people. Our review has three major findings. First, longitudinal trajectory-based studies found two to five positive and negative symptoms trajectories in patients and four to six cognitive trajectories in patients, siblings, controls, or combined samples. Second, cross-sectional cluster-based studies identified three positive and negative symptoms clusters among patients and four positive and negative schizotypy clusters among healthy siblings. Additionally, three to five cognitive clusters were discovered in patients and their unaffected relatives. Third, numerous sociodemographic, clinical and genetic factors that determine trajectories and/or clusters were identified.

We showed that longitudinal and cross-sectional studies in patients, their siblings and healthy general population have inconsistently identified two to five trajectories/clusters and various predictors across the schizophrenia symptoms and cognitive deficits. Several shortcomings across studies may cause this inconsistency. Previous longitudinal studies did not uniformly research symptoms and cognitive deficits. For example, only three studies^16,52,66^ longitudinally investigated cognitive trajectories, but 22 cross-sectional studies investigated cognitive clusters. Utterly, none of the reviewed longitudinal and cross-sectional studies also validated their model using empirical methods or comparable statistical methods though they have used different complex data-driven methods. Accumulating evidence showed that the number of classes in the optimal model derived from one method can be remarkably different compared to the other method^96^. Given that these studies were conducted in patients at a different stage at diagnosis, disease course or severity of illness and treatment status, the results may not be expectedly consistent as well. For example, studies that included only first-episode psychosis, chronic or stable patients may identify smaller clusters than studies that included a mixture of patients with first-episode and chronic psychosis or patients with severe illness. Additionally, since the reported studies were conducted in more than 20 countries, the use of different treatment strategies and assessment methods in different countries could further confound the assessment of symptoms and clinical heterogeneity. Obviously, in patients who are treated, the observed symptoms and cognitive characteristics are the product of those features that were present before treatment and the response to treatment. Moreover, the different measurement tools may lead to discrepant results. For instance, the discrepancy of negative and positive symptoms trajectories (or cross-sectional clusters) might partly be attributable to the use of a specific negative (e.g. SANS) and positive (e.g. SAPS) symptom scale or a more general symptom scale (e.g. PANSS) that included items measuring cognitive or disorganization symptoms. Additionally, some studies administered up to 18 different neuropsychological tests to measure cognition while others have used as few as two or three cognitive assessment tests.

We further observed common methodological limitations across studies. Firstly, the reviewed studies included various groups of participants from different age groups and ethnicities. Secondly, while the comparison of patient clusters and trajectories with healthy siblings or controls could provide an accurate means of disentangling the heterogeneity and causes of heterogeneity of schizophrenia symptoms, only four studies (three were cross-sectional studies) examined clusters in siblings. Likewise, most studies used healthy controls to standardize patients neurocognitive composite scores, and a few other studies used controls to compare the distribution of patient clusters or trajectory groups. Thirdly, substantial differences between studies were also noted in constructing composite scores, use of model selection criteria and method of parameter estimation. Fourthly, we observed several ways of subtyping and nomenclature for clusters or trajectories, which may be difficult for clinicians to translate the evidence in diagnosing and treating diseases. This is due to the lack of a standard for designing a study (e.g. adequate sample size), reporting data analysis approaches and publishing results^42^.

Generally, we saw that studies conducted in patients with similar stages of illness (i.e, first-episode, stable, chronic stage or with or without treatment) and used similar assessment methods (i.e., SANS, SAPS or PANSS) showed some level of similarity in results with respect to identified trajectories and predictors, but studies are largely different in duration of follow-up, frequency of assessment and methods used to assess symptoms or cognition. By the same token, studies that used the similar data-driven statistical methods showed similarity in the number of identified trajectories/clusters, but largely different in study population, stage of illness, use of measurement tool, duration of follow-up, frequency of assessment and identified factors. Moreover, studies with duration of follow-up less than two years and above two years showed a similar level of heterogeneity in symptoms and cognitive deficits and identified predictors. In addition, a 10-year study with five times assessment showed similar findings with a 6 week study with every week assessment on positive symptoms. On the other hand, a 2-year study with five times assessment identified only two trajectories. Despite these facts, all studies interestingly showed heterogeneity of symptoms and cognitive deficits at various level with “four trajectories” is the most replicated in longitudinal studies and “three clusters” is the most replicated in cross-sectional studies. Besides, these studies consistently reported age, gender, ethnicity, educational status, age of illness onset, diagnosis, general psychopathology and depressive symptoms, positive and negative symptoms, cognitive performance, functioning and quality of life as determinant factors of trajectories and/or clusters.

**Fig. 4:**
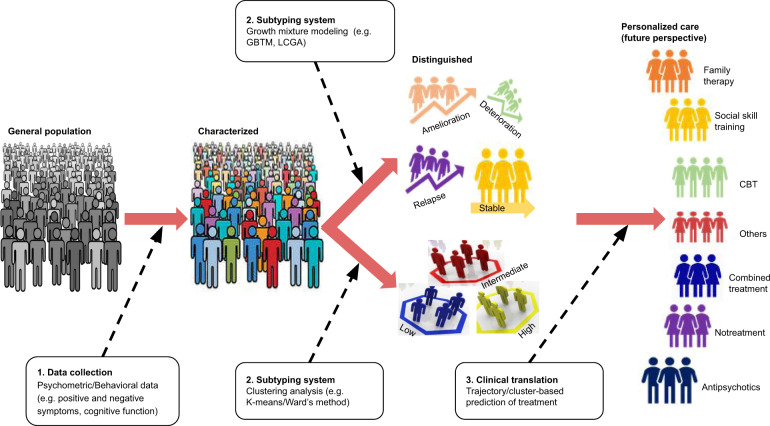
A hypothetical model for driving big multidimensional data towards a personalized selection of treatments in schizophrenia spectrum disorders. GBTM: Group-based trajectory modeling; LCGA: Latent class growth analysis; CBT: Cognitive behavioural therapy.

In the era of team science and big data, the use of data-driven statistical methods is becoming increasingly popular for the analysis of longitudinal repeated measures (i.e., latent growth mixture models (LGMMs)) and cross-sectional (i.e., cluster analysis) data (Fig. 4). In our review, we observed that LGMMs, such as GMM, latent class growth analysis (LCGA), mixed mode latent class regression modelling and group-based trajectory modelling (GBTM) were commonly used data-driven methods in longitudinal studies. LGMMs can identify realistic categories based on temporal patterns of change in outcome by assuming the existence of latent classes or subgroups of subjects exhibiting similarity with regard to unobserved (latent) variables^19,97^. LGMMs have four advantages for modelling longitudinal data. First, they are flexible and data-driven methods that can accurately reveal actual heterogeneity. Second, they allow the classification of individual subjects into latent classes based on the largest probability of class membership. Third, they are sensitive to the pattern of change over time and robust in the presence of missing data. Fourth, subject-level predictors can be directly assessed for association with class membership and hence with different trajectory subtypes^16,19,97^. Cluster analysis, which is commonly used in cross-sectional studies, is also a data-driven approach for classifying people into homogeneous groups by determining clusters of participants that display less within-cluster variation relative to the between-cluster variation^81^. Among the reviewed cross-sectional studies, K-means and Ward’s method clustering analyses were commonly used alone or in combination. K-means cluster analysis is a non-hierarchical form of cluster analysis appropriate when previous evidence or hypotheses exist regarding the number of clusters in a sample^74^. On the other hand, Ward’s method is a hierarchical cluster analysis aiming to determine group assignment without prior hypothesis^74^. It is believed, K-means cluster analysis can handle larger data sets compared with Ward’s method^73^.

The results of statistical subtyping approaches, such as cluster or trajectory analysis depend on mathematical assumptions, type of data, number of variables or tests, sample size and sampling characteristics. Therefore, the models can be unstable and parameter estimates of clinical symptoms may not converge to a consistent set of subgroups and lack a direct relationship to clinical reality^59,87,98^. For example, intermediate clusters and trajectories substantially vary between studies that used the same cluster or trajectory analysis method^87^. We advocate that study results from data-driven methods should be applicable, comparable, generalizable and interpretable into clinical practice. As a result, we recommend to validate models using at least one additional comparable statistical methods, combine statistical methods of subtyping with empirical/clinical methods, or work together with clinicians to create a common understanding and clinically relevant clustering or trajectories nomenclatures. Furthermore, it is relevant to replicate clusters or trajectory groups using independent samples, different assessment tools that measure the same construct, or different linkage methods^38,99^. Finally, further studies are required that focus on longitudinal study design, unaffected siblings, genetic markers and more detailed measures of brain network function for improving our understanding of the biological mechanism underlying heterogeneity of schizophrenia.

Future clinical advances may benefit from the subgrouping of patients to implement tailored therapy. In our review, we observed that several longitudinal studies were conducted based on drug response. One study found individuals who treated with aripiprazole had delayed response^56^, whereas another study found olanzapine treated patients had good response^63^. Another study also revealed individuals receiving standard treatment, compared to assertive treatment, showed delayed negative symptom trajectory^19^. Furthermore, individuals with substantial cognitive deficit received high dose of antipsychotics^18,82,87^. Subtyping of symptoms and cognitive deficits can also contribute to uncover the biological basis of individual symptoms, rather than studying constellation of co-occurring symptoms^1^. The identified factors associated with clusters and/or trajectories could be used for developing a clinical risk prediction model for high-risk individuals with prodromal symptoms^100,101^.

Thus far, findings from this review showed that data-driven approaches could have substantial role to optimize the efficacy of personalized care by predicting individual susceptibility to disease, providing accurate assessments of disease course, contribute to best-choice of early intervention, and selecting treatments (e.g., antipsychotics, cognitive behavioral therapy, social skill training, family therapy) targeting subgroups of patients with similar phenotypic or psychosocial characteristics (Fig. 4)^102^. When data-driven methods are implemented on samples/cohorts following different pharmacological and non-pharmacological interventions, then, we believe that our proposed model (Fig. 4) can identify individuals who successfully treated, not treated or even harmed and who needs further intervention and close follow-up to protect from unnecessary cost and side effect of medication(s). Therefore, findings from our review could assist in the implementation of personalized and preventive strategies for clinical practice at least in national or regional level.

## Conclusions

Our review indicated a significant heterogeneity in results and conclusions obtained from both cross-sectional and longitudinal studies in terms of the number of group membership for positive and negative symptoms and cognition as well as factors (predictors) associated with the group membership. This review also identified several methodological issues contributing to the discrepant results. Generally, the longitudinal studies identified trajectories characterized by progressive deterioration, relapsing, progressive amelioration and stability, whereas low, mixed (intermediate) and high psychotic symptoms and cognitive clusters were identified by cross-sectional studies. Future studies can be more benefited from data-driven methods if applied based on pharmacological and non-pharmacological treatment responses. The use of empirical methods to distinguish more homogeneous subgroups of patients along heterogeneous symptom dimensions has gained traction in the last several years and it is an essential step toward implementation of a more precise prediction of disease risk and individualized selection of interventions.

## Supplementary information

Supplementary information

## Data Availability

All relevant data were included in the paper.
