# Supplementary material for: A systematic review and narrative synthesis of data-driven studies in schizophrenia symptoms and cognitive deficits
Source: Transl Psychiatry. 2020 Jul 21;10:244. doi: 10.1038/s41398-020-00919-x (PMC7374614; doi:10.1038/s41398-020-00919-x)
Supplement: Supplementary file 1 — Supplementary information [file 41398_2020_919_MOESM1_ESM.docx]

**PsycINFO**

| Search | Query | Number of hits |
| --- | --- | --- |
| #1 | MA Schizophrenia OR AB Psychosis OR AB Schizophren* OR AB Psychoses OR AB Psychotic | 154,968 |
| #2 | MA Cognitive Impairment OR MA Cognition OR MA Cognitions OR MA Cognitive Dysfunction OR MA Social Amotivation OR MA Expressive Deficit OR MA Symptom* OR AB Cognitive Ability OR AB Cognitive OR AB Neurocognit* OR AB Psychopathol* OR AB deterioration* OR AB Neuropsychologic* OR AB endophenotyp* | 457,697 |
| #3 | MA Latent Class OR MA Cluster Analysis OR MA Subtype* OR MA Trajector* OR AB Subgroup* OR AB Cluster* OR AB Profile OR AB Heterogeneity | 171,658 |
| #4 | #1 AND #2 AND #3  Limiters - Publication Year: 2008-2018; Language: English; Full Text; Document Type: Journal Article  Search modes - Find all my search terms | 3,544  251 |

**PsycARTICLES**

| Search | Query | Number of hits |
| --- | --- | --- |
| #1 | MA Schizophrenia OR AB Psychosis OR AB Schizophren* OR AB Psychoses OR AB Psychotic | 5,951 |
| #2 | MA Cognitive Impairment OR MA Cognition OR MA Cognitions OR MA Cognitive Dysfunction OR MA Social Amotivation OR MA Expressive Deficit OR MA Symptom* OR AB Cognitive Ability OR AB Cognitive OR AB Neurocognit* OR AB Psychopathol* OR AB deterioration* OR AB Neuropsychologic* OR AB endophenotyp* | 21,881 |
| #3 | MA Latent Class OR MA Cluster Analysis OR MA Subtype* OR MA Trajector* OR AB Subgroup* OR AB Cluster* OR AB Profile OR AB Heterogeneity | 5,933 |
| #4 | #1 AND #2 AND #3  Limiters - Year of Publication: 2008-2018; Full Text; Document Type: Journal Article  Search modes - Find all my search terms | 144  27 |

**PubMed**

| Search | Query | Number of hits |
| --- | --- | --- |
| #1 | ("Schizophrenia Spectrum and Other Psychotic Disorders"[Mesh] OR schizophren*[tiab] OR psychosis[tiab] OR psychoses[tiab] OR psychotic[tiab]) | 188,707 |
| #2 | ("Cognition Disorders"[Mesh:NoExp] OR "Cognitive Dysfunction"[Mesh] OR "Cognition"[Mesh] OR cognition[tiab] OR cognitive[tiab] OR neurocognit*[tiab] OR symptom*[tiab] OR deficit syndrome*[tiab] OR psychopathol*[tiab] OR deterioration*[tiab] OR (social[tiab] AND amotivation[tiab]) OR (expressive[tiab] AND deficit*[tiab]) OR neuropsychologic*[tiab] OR endophenotyp*[tiab]) | 1,448,910 |
| #3 | ((( subgroup[tiab] OR subtype* [tiab] OR cluster [tiab] OR groups[tiab] OR profile[tiab]) AND ((cluster[tiab] AND analysis[tiab]) OR trajectory[tiab] OR trajectories[tiab] OR (latent[tiab] AND class[tiab] AND analysis[tiab]) OR heterogenity[tiab])) | 98,557 |
| #4 | #1 AND #2 AND #3  Limiters - Publication Year: 2008-2018; Language: English; Full Text; Document Type: Journal Article | 801  522 |

**SCOPUS**

| Search | Query | Number of hits |
| --- | --- | --- |
| #1 | TITLE-ABS-KEY ( schizophrenia ) OR TITLE-ABS-KEY ( psychosis ) OR ABS ( schizophren* ) OR ABS ( psychoses ) OR ABS ( psychotic ) | 264,841 |
| #2 | TITLE-ABS-KEY ( cognition AND disorders ) OR TITLE-ABS-KEY ( cognitive AND impairment ) OR ABS ( cognitive AND dysfunction ) OR ABS ( cognition ) OR ABS ( cognitions ) OR ABS ( social AND amotivation ) OR ABS ( expressive AND deficit ) OR ABS ( symptom* ) OR ABS ( cognitive AND ability ) OR ABS ( cognitive ) OR ABS ( neurocognit* ) OR ABS ( psychopathol* ) OR ABS ( deterioration* ) OR ABS ( neuropsychologic* ) OR ABS ( endophenotyp* ) | 1,897,943 |
| #3 | ( ( ABS ( subgroup ) OR ABS ( subtype* ) OR ABS ( cluster ) OR ABS ( groups ) OR ABS ( profile ) ) ) AND ( ( ABS ( cluster AND analysis ) OR ABS ( trajectory ) OR ABS ( trajectories ) OR ABS ( latent AND class AND analysis ) OR ABS ( heterogeneity ) ) ) | 301,589 |
| #4 | #1 AND #2 AND #3  Limiters - Publication Year: 2008-2018; Language: English; Document Type: Article and Article in press | 1,542  715 |

**EMBASE**

| Search | Query | Number of hits |
| --- | --- | --- |
| #1 | 'schizophrenia spectrum disorder'/exp OR 'psychosis'/exp OR 'psychoses':ab,ti OR 'psychotic':ab,ti OR 'schizophren*':ab,ti | 300,286 |
| #2 | 'cognitive defect'/exp OR 'cognition'/mj OR 'cognition':ab,ti OR 'cognitive':ab,ti OR 'deficit schizophrenia':ab,ti OR 'neurocognit*':ab,ti OR 'symptom*':ab,ti OR 'psychopathol*':ab,ti OR 'social amotivation':ab,ti OR 'expressive deficit*':ab,ti OR 'neuropsychologic*':ab,ti OR 'endophenotyp*':ab,ti | 2,084,416 |
| #3 | (('subgroup':ab,ti OR 'subtype':ab,ti OR 'cluster':ab,ti OR 'groups':ab,ti OR 'profile':ab,ti) AND ('cluster analysis':ab,ti OR 'trajectory':ab,ti OR 'trajectories':ab,ti OR 'latent class analysis':ab,ti OR 'heterogeneity':ab,ti)) | 70,195 |
| #4 | #1 AND #2 AND #3  Limiters - Publication Year: 2008-2018; Language: English; Document Type: Journal Article | 1,271  389 |

**Web of Science**

| Search | Query | Number of hits |
| --- | --- | --- |
| #1 | TS=(schizophrenia) OR TI=(schizophren*) OR TS=(psychosis) OR TI=(psychoses) OR TI=(psychotic) | 205,758 |
| #2 | TS=(Cognition Disorders) OR TS=(Cognitive Dysfunction) OR TS=(Cognitive decline) OR TS=(Cognitive deficit) OR TS=(Cognitive impairment) OR TS=(Cognition) OR TS=(Disease Progression) OR TI=(cognition) OR TI=(cognitive) OR TI=(neurocognit*) OR TI=(symptom*) OR TI=(deficit syndrome*) OR TI=(psychopathol*) OR TI=(progression*) OR TI=(deterioration*) OR TI=(social amotivation) OR TI=(expressive deficit*) OR TI=(neuropsychologic*) OR TI=(endophenotyp*) | 795,682 |
| #3 | TI=(subtype*) OR TI=(cluster*) OR TI=(profil*) OR TI=(traject*) OR TI=(trajector*) OR TI=(latent class analy*) OR TI=(heterogeneity) | 543,555 |
| #4 | #1 AND #2 AND #3  Limiters - Publication Year: 2008-2018; Language: English; Document Type: Article | 937  358 |
